# Compassionate Work Environments and Their Role in Teachers’ Life Satisfaction: The Contribution of Perceived Collective School Performance and Burnout

**DOI:** 10.3390/ijerph192114206

**Published:** 2022-10-30

**Authors:** Ilaria Buonomo, Martina Pansini, Sara Cervai, Paula Benevene

**Affiliations:** 1 Department of Human Sciences, LUMSA University, 00193 Rome, Italy; 2 Department of Political and Social Sciences, University of Trieste, 34127 Trieste, Italy

**Keywords:** burnout, compassion, school, teachers, life satisfaction, work well-being

## Abstract

Several studies on helping professionals showed the protective role of compassion among colleagues and leaders. Despite this, studies on well-being factors at school, both preventive and protective, usually focus on teachers’ personal resources and study compassion in the teacher–student relationship. This study explores the role of received compassion at work on teachers’ life satisfaction while considering perceived school collective performance and burnout conditions as mediators in this link. One hundred and eighty-six Italian teachers (female = 85.4%, mean age = 48.5, SD = 9.46) completed a questionnaire on received compassion at work, perceived school collective performance, burnout, and life satisfaction. Through a structural equation model (χ^2^(21) = 30.716, *p* = 0.08, CFI = 0.989, TLI = 0.981, RMSEA = 0.050 (90% CI = 0.000–0.080, *p* = 0.465), SRMR = 0.038), it emerged that only perceived school collective performance mediated the association between received compassion and life satisfaction. To the best of our knowledge, few studies have addressed the role of compassion received from colleagues and supervisors at school and its effect on teachers’ work-related beliefs and personal well-being.

## 1. Introduction

International reports and studies describe teachers’ daily challenges at work (e.g., technology use, multiculturality, special educational needs, oppositive behaviors, and normative changes) [1,2,3]. Furthermore, according to the European Trade Union Committee for Education, teachers are at the highest level of work-related stress in several European countries [4]. Research usually highlights the protective role of teachers’ personal resources in dealing with such challenges: pedagogical skills, classroom management abilities, efficacy and preparedness beliefs, and stress management techniques [5,6,7]. These dimensions are crucial for teaching-learning processes [8,9] and promote teachers’ personal well-being [10,11]. 

At the same time, teaching tasks and conditions encompass several dimensions, i.e., the teacher–student relationship, the teacher–school relationship, and teacher relationships with colleagues and the principal, that may benefit from implementing different resources. Such relational complexity, indeed, may heighten the risk of harmful health conditions. Nevertheless, the web of potentially harming interactions may play a positive role if adequately addressed and strategically used. The high complexity of today’s schools, indeed, calls for a substantial take for teachers, principals, and administrative staff members to act as educational communities where each member can generate and access collective resources, starting from collaborative processes and practices. Schools may benefit from the experiences of other helping professionals’ communities by structuring an effective collaborative system. This system may allow school members, from one side, to adequately manage the challenges and, from the other, to share the related cognitive and emotional load [12,13,14,15]. Overall, these processes may empower and enrich all school staff members instead of harming them.

Among the constructs that, in the organizational psychology literature, account for the onset of such practices, compassion has been shown to have several advantages for different professionals [16,17,18,19,20]. Furthermore, studies on compassion at work reported positive effects above and beyond the workplace, showing that receiving compassion boosts mental health and happiness across life domains [21]. 

However, the role of compassion received at work has mainly been analyzed in social and healthcare contexts. Limited research investigated the role of compassion in the educational field, despite it being a caring context. This research aims to fill this gap. Consequently, findings from previous studies on the teaching profession even supplemented with those provided by studies on other professional categories, drove the building of the hypotheses, as shown below.

### 1.1. The Benefits of Compassionate Workplaces

Compassion includes three abilities: noticing others’ suffering, feeling the urge to do something to reduce their pain or prevent future pain, and taking effective actions [22]. The focus on suffering, the urge to help, and the action taken at the advantage of others make compassion different from other constructs describing the ability to tune in with others’ emotions, such as empathy [22]. 

The theme of compassion within schools is not new. As helping professionals, teachers are urged to show compassion toward their students [23,24,25,26]. However, while this ability is a part of teaching skills, it is mainly confined to the teacher-student interaction, neglecting its potential effects when oriented towards colleagues in times of stress and struggling at work.

Suffering, indeed, is a common work condition [27] affecting negative emotions, stress, or burnout conditions [27,28,29]. Common sources of suffering at work include personal events [29], poor relationships at work [30], organizational changes [31], or the job role itself [31], above all, when implying caregiving tasks [32]. Thus, it is likely that teachers may have access to suffering-inducing conditions at work. 

Receiving compassion from others at work was linked to several advantages, including higher job satisfaction and organizational commitment, higher person-organization fit, and higher psychological safety [27,28,33,34].

Employees can receive compassion at work from three different sources: colleagues, superiors, and the organization at large [29]. 

Interestingly, compassion boosts a sense of community and of belonging to the organization. It is related to the idea that there is a supportive network within the workplace to share the load of job demands and notice when one is suffering. In this regard, research has shown the positive effect of receiving compassion at work on burnout among healthcare professionals [35] and public service employees [36]. In the first case [35], compassion to colleagues was negatively related to depersonalization and emotional exhaustion and positively associated with professional accomplishment. In the second one, an association was found between receiving experienced compassion from one’s supervisor and having a lower risk of burnout in a six month time frame in a sample of public service employees [36]. Thus, these being highly relational and caring professions, this association could also be confirmed in teachers. 

Even when social support is not operationalized as compassion, such supportive dynamics were previously linked with stress and burnout prevention in the educational context. According to the JDR model [37] social support is an effective coping strategy and a preventive factor for the detrimental impact of job demands on burnout onset (e.g., [38]). In this regard, the greater the workplace support from colleagues and supervisors, the lower the burnout experienced by teachers, even when compared to family support [39]. Social support also has a protective role for teachers and principals’ well-being. Effective sources of emotional and instrumental support inside and outside the workplace prevents adverse psychological conditions and promotes well-being and performance for these professionals [39,40,41]. Overall, receiving support and compassion at work acts a job resource, as it may support the achievement of goals, reduce job demands and associated strains, or foster professional growth [37]. For these reasons, we aim to verify that:

**H1.**  *Compassion experienced at work is negatively related to teacher burnout.*

Compassion shapes employees’ representations of themselves, colleagues, organizations, and the job experience in general [16,29,33,42]. This link is consistent with Fredrickson’s Broaden and Build theory of positive emotions [43]. In this theory, when people acknowledge positive emotions in their daily life, they can broaden their cognitive and behavioral repertoire in the short term and build personal resources in the long term. Consistently, when employees experience compassion, they build a positive work identity which they are motivated to reinforce to maintain a sense of personal value over time; this process leads to deploying commitment toward the organization [44]. Furthermore, when compassion is spread throughout the organization, constituting a key value in its culture, it promotes a dimension of collectivity, trust, and interconnectedness among its members. In this perspective, employees perceive the organization as a source of care and support and develop a positive collective work identity. As found, employees increase the quality of their job performance to enforce these beliefs further and contribute to organizational objectives [45,46]. Similarly, frequent compassionate experiences in the workplace lead teachers to make positive emotional associations with the workplace and express positive attitudes toward the organization. Such attitudes are expressed in emotional vigor, job satisfaction, organizational commitment [29,33], and work engagement [47]. 

Such positive representations regard perceived performance as well. For example, receiving compassion is positively associated with perceived individual performance in teachers [16] and other professional categories [28,36,45]. 

As stated above, the teaching profession is inherently relational, as it is pursued within an intricate web of relationships with administrators, principals, colleagues, students, and their families. For this reason, other than individual performance, it is helpful to study teachers’ representations of collective performance. Within the teaching profession, indeed, it was shown that perceiving a sense of collectivity at school boosts professional well-being and effectiveness [48,49,50]. Furthermore, among constructs tackling this type of representation, perceived collective performance [51] is a valuable indicator of the degree to which workers acknowledge the quality and quantity of work outcomes and their contribution to them.

Literature on healthcare workers showed that compassionate workplaces boost positive representations of organizations as communities [19]. Although this connection was overlooked for other helping professionals (teachers included), compassion may help clarify the impact of caring skills from the relationship with users to the interactions with colleagues. Therefore, building on this literature, we aim to verify whether:

**H2.**  *Compassion experienced at work is positively related to perceived school collective performance.*

Compassion at work benefits personal well-being as well. It is linked with improvements in positive affects [29,33,42], psychological well-being [52], and perceived general health [21,53]. These outcomes are consistent with the idea that life satisfaction emerges from several life domains [54]. In other words, the higher the satisfaction with work, family, health, and leisure, the higher the perceived satisfaction with life in general. Regarding the work domain, life satisfaction is impacted by job characteristics, such as job-related tensions and quality of work-life, the least including supportive work environments [55]. While some studies underlined the effects of job-related variables on teacher life satisfaction [56,57], no studies, to the best of our knowledge, addressed this link while including compassionate relationships as an antecedent. Such a link was not studied in other professional fields either. At the same time, according to the theory of subjective well-being, life satisfaction is a cognitive dimension of general well-being [58]. Thus, it may be helpful to consider studies that, despite not addressing the effects of compassion on life satisfaction, account for its effects on general mental health outcomes. In this regard, healthcare practitioners’ studies showed that the higher the compassion experienced at work, the higher the general well-being [59]. 

Thus, we aim to verify whether:

**H3.**  *Compassion experienced at work is positively related to teachers’ life satisfaction.*

### 1.2. Linking Work Experiences and Life Satisfaction

Considering the effects of job-related variables on life satisfaction, it is apparent that the perceptions of one’s job and organization have a role in influencing subjective well-being [55]. As mentioned, latter last is considered a multifaced construct including life satisfaction, job satisfaction, positive affect, and absence of job stress or negative affect at work [58]. With this regard, a meta-analysis showed that organizational performance and commitment significantly correlate with general well-being [55]. This is true for teachers as well. For example, using the JD-R model [37] as a theoretical framework, Granziera and colleagues [60] reviewed research in which job resources predicted teachers’ efficacy, well-being, and satisfaction. Moreover, hedonic balance (the emotional counterpart of life satisfaction in Diener’s subjective well-being construct) was positively correlated with teachers’ sense of collective efficacy [48]. With specific regard to the variables inserted in this study, to the best of our knowledge, only one study addressed the relationship between collective performance and subjective well-being in teaching, showing a correlation between the two [61]. Overall, building on the few contributions on teachers, together with literature on other professionals, we expect that:

**H4.**  *Perceived school collective performance has a positive association with teachers’ life satisfaction.*

Similarly, chronic stress conditions at work have been linked to life satisfaction. Several studies showed that burnout symptoms are detrimental to workers’ physical and mental health [62,63,64], and the teaching profession makes no exception [8,65]. Traditionally, the literature suggests that teachers are one of the most vulnerable categories at risk of burnout [66]. Burnout is a negative work-related syndrome emerging from the repeated exposure to stressful events at work, which core symptoms are emotional exhaustion, cynicism, and depersonalization and sense of ineffectiveness [67]. Several studies have been describing the potential detrimental effects of job demands at school, building on the JD-R model [67]. Furthermore, it was shown that teacher burnout can lead to negative outcomes outside the school as well. For example, teachers’ levels of burnout negatively affect their mental well-being [67], the process by which they appraise school-life events, their negative emotional intensity [68], and their private life [69]. In line with previous results (e.g., [70]), Pelaez-Fernandez and colleagues [71] found that burnout dimensions such as emotional exhaustion and depersonalization were related negatively to life satisfaction. However, the personal accomplishment was positively associated with life satisfaction in Spanish teachers. Thus, we expect that:

**H5.**  *Burnout has a negative association with teachers’ life satisfaction.*

### 1.3. Compassion, Work Experiences, and Life Satisfaction

The mentioned studies showed that compassionate relationships at school, teacher-perceived collective performance, and burnout can affect teachers’ life satisfaction. At the same time, compassionate relationships prevent burnout and promote a positive perception of one’s organization. Thus, these dimensions may mediate the associations between received compassion and life satisfaction. Regarding perceived collective performance, indeed, the positive contribution of compassion in teachers’ perceptions about the school performance can influence their general satisfaction. This may confirm the idea behind the Conservation of Resources theory (COR) [72], according to which life domains are interrelated, and individuals can benefit from resources built in one domain (e.g., work) even when they are not interacting with it. Similarly, the COR theory states that workers may lose resources because of the permeability across life domains: high levels of burnout may likely disrupt the link between received compassion and life satisfaction. However, to the best of our knowledge, no study addressed the role of received compassion in these links, while some studies (e.g., [73,74]) addressed the role of self-compassion, showing that professional quality of life positively affects personal well-being [73] and even that engagement and resilience at work are significantly related to a sense of satisfying and purposeful life [74]. These results highlight how the self-efficacy experienced at work positively affects employees’ well-being outside of work. At the same time, considering the solid theoretical underpinnings of the COR theory and the findings on self-compassion, we can expect that:

**H6.**  *Perceived school collective performance and burnout mediate the relationship between experienced compassion and life satisfaction.*

**Figure 1:**
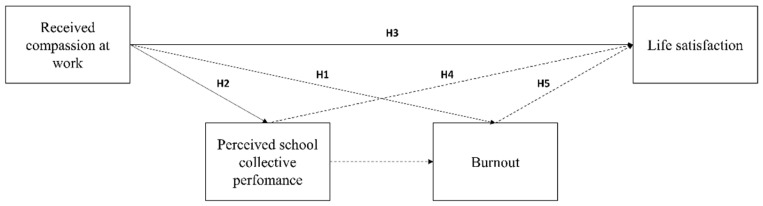
Theoretical model and hypotheses.

Figure 1 synthesizes and represents all the mentioned hypotheses. 

## 2. Materials and Methods

### 2.1. Participants 

Participants were 186 (female = 85.4%) Italian teachers, aged 36–65 years (M = 48.49, SD = 7.48). About 72% had a university degree, while 30% had an upper secondary school diploma. Nearly 49% of the teachers taught in a primary school, while 51% in a secondary school. Teachers had 0–36 years of experience (M = 11.73, SD = 9.46), and 89% of them had an open-ended contract.

Participants signed an informed consent form providing information about the confidentiality and anonymity of the administration and the independence of the research group from their school administrators. In addition, it was stated that only the researchers could access the data and that no data report would be given to the school management. Overall, these procedures would address a potential social desirability bias.

### 2.2. Measures 

A questionnaire composed of four measures was administered to the participants. For each measure, Cronbach’s alpha, compositive reliability, and McDonald’s omega scores were used to provide information about internal consistency. Cronbach’s alpha scores should be at least equal to 0.70 [75], and composite reliability and McDonald’s omega scores should be at least 0.80 [76,77] to indicate adequate reliability. 

Experienced compassion at work was measured through the experienced compassion at work scale [29]. It includes three items measured on a 5-point Likert scale (1 = “Never” and 5 = “Nearly all the time”). A sample item is: “How often have you experienced compassion at work?”. Cronbach’s alpha in this study is 0.831, while CR value is 0.903, and McDonald’s omega is 0.846.

Perceived collective school performance was measured through the organizational-level performance scale [51]. It includes six items indicating organizational outcomes, measured on a 5-point Likert scale (1 = “Very poor” and 5 = “Very good”). Items were adapted to fit the teachers’ working experiences. For example, original items including “products and services,” were modified to “services” only. A sample item is: “Relationship between administration and employees”. Cronbach’s alpha in this study is 0.951, while CR value is 0.970, and McDonald’s omega is 0.951. 

Burnout was measured through the professional quality of life scale [78]. It includes ten items measured on a 5-point Likert scale (1 = “Never” and 5 = “Very often”). A sample item is: “I feel trapped in my job as a teacher”. Cronbach’s alpha in this study is 0.787, while CR value is 0.836, and McDonald’s omega is 0.745.

Life satisfaction was measured through the satisfaction with life scale [79]. It includes five items measured on a 7-point Likert scale (1 = “Strongly disagree” and 7 = “Strongly agree”). A sample item is: “In most ways, my life is close to my ideal”. Cronbach’s alpha in this study is 0.901, while CR value is 0.930, and McDonald’s omega is 0.907.

Overall, the questionnaire included 24 items, administered to 186 subjects, thus respecting the most commonly used subject-variables ratios, such as the 5:1 ratio [80,81], the rule of 100 [82], the 3–6 ratio [83]. 

### 2.3. Plan of Analyses

Firstly, we explored the data with three procedures: (a) detecting univariate and multivariate outliers using the Mahalanobis’s distance method, set to *p* < 0.001 [84]; (b) analyzing score distribution, setting skewness and kurtosis cut-off points to [−2; +2] [85]; and (c) analyzing missing values and omitting them listwise [86]. After these procedures, we deleted 17 subjects and obtained the sample described in the Participant section. The number is adequate according to current indications in methodology research on SEM models [87,88,89].

Secondly, we tested the common method variance bias using Harman’s single-factor test. The single factor emerging from the exploratory factor analysis only accounted for 36% of the covariance among the measures, showing no issues associated with this bias [90]. 

Thirdly, Pearson’s correlations were measured among burnout, life satisfaction, perceived collective school performance, and experienced compassion, to verify the associations between the variables and such constructs and demographics (age, gender) and work-related variables (experience, primary vs. secondary school).

After the preliminary analyses with IBM-SPSS v. 24, a confirmatory factor analysis (CFA) [91] was performed to examine the measurement model with MPlus version 8 [87]. Two item parcels were created for the administered measures to enhance our model’s reliability and parsimony. Each parcel was created by sequentially summing items assigned based on the highest to lowest item-total corrected correlations [92,93,94]. Parceling reduces free parameters to estimate and sampling error sources. Because the compassion measure includes only three items, parceling was not implemented for this scale. The robust maximum likelihood approach (MLR) was used to deal with non-normality in data [95]. To test the validity of the measures used in the model, average variance extracted (AVE) measure was performed. AVE higher than 0.50 indicates a good convergent validity [96]. 

Next, a structural equation model (SEM) [91] was implemented. Under the model, experienced compassion was directly and indirectly (through perceived school collective performance and burnout) associated with life satisfaction. We followed a multifaceted approach to the assessment of the fit of the model [97], using the following indices: the Chi-square likelihood ratio statistic, the Tucker and Lewis index (TLI), the comparative fit index (CFI), the root mean square error of approximation (RMSEA), and the standardized root mean square residual (SRMR). We accepted TLI and CFI values greater than 0.95 [98], RMSEA values lower than 0.08 [99,100], and SRMR values lower than 0.08 [98,100].

## 3. Results

### 3.1. Measurement Model and Correlations among the Variables

The CFA showed a good fit to the data: χ^2^(21) = 30.716, *p* = 0.08, CFI = 0.989, TLI = 0.981, RMSEA = 0.050 (90% CI = 0.000–0.080, *p* = 0.465), SRMR = 0.038. AVE scores were 0.650 for experienced compassion, 0.908 for perceived school collective performance, 0.667 for burnout, and 0.832 for life satisfaction. 

Table 1 shows the correlations among experienced compassion, burnout, perceived school collective performance, and life satisfaction. As expected, experienced compassion showed a positive correlation with perceived school collective performance (r = 0.564, *p* < 0.01) and life satisfaction (r = 0.473, *p* < 0.01), and a negative correlation with burnout (r = −0.324, *p* < 0.01). Furthermore, life satisfaction showed a positive association with perceived school collective performance (r = 0.547, *p* < 0.01) and a negative association with burnout (r = −0.440, *p* < 0.01). Correlations among such variables and demographic and work-related variables are not shown, as they were not significant (*p* > 0.05). 

### 3.2. Final Model 

**Figure 2:**
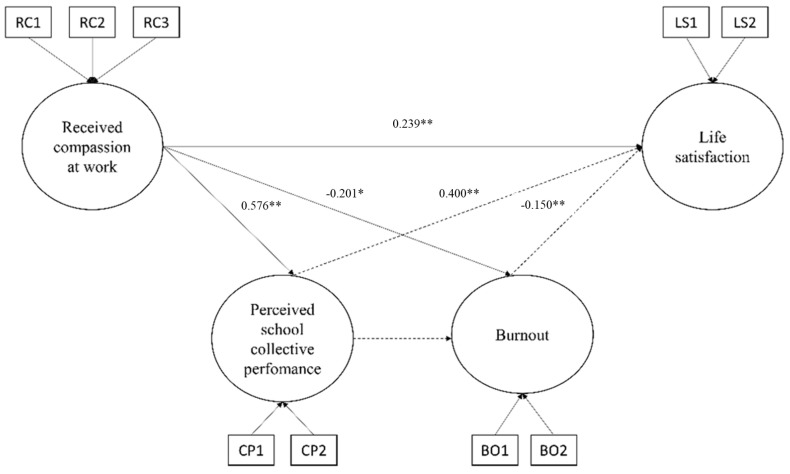
Results of the structural equation model. * = *p* < 0.05, ** = *p* < 0.01.

The SEM model (Figure 2), showed a good fit to the data χ^2^(21) = 30.716, *p* = 0.08, CFI = 0.989, TLI = 0.981, RMSEA = 0.050 (90% CI = 0.000–0.080, *p* = 0.465), SRMR = 0.038. Experienced compassion was positively associated with perceived school collective performance (b = 0.576, *p* < 0.001) and burnout (b = −0.201, *p* < 0.05), confirming H1 and H2. Furthermore, perceived school collective performance was positively correlated to life satisfaction (H4; b = 0.400, *p* < 0.001), as well as burnout (H5; b = −0.150, *p* < 0.001), confirming H4 and H5. 

The direct association between experienced compassion and life satisfaction (H3) was confirmed. (b_DIRECT_ =0.239, *p* = 0.007). At the same time, while perceived school collective performance partially mediated the relationship between experienced compassion and life satisfaction (b_INDIRECT_ = 0.230, *p* = 0.000), burnout did not mediate such an association (b_INDIRECT_ = ns). Thus, H6 was only partially confirmed. 

The percentages of variance explained were 33.2% for perceived school collective performance, 17.2% for burnout, and 42% for life satisfaction.

## 4. Discussion

The findings showed an association between experienced compassion and life satisfaction. Perceived school collective performance partially mediated this link, while burnout did not. 

Regarding the association between experienced compassion and life satisfaction, our findings confirm positive relationships’ role in building well-being conditions. This link, indeed, is consistent with the research on compassion and its effect on psychological well-being and positive affects [42,101,102]. Furthermore, it can be understood in light of the COR theory [72]. This approach states that life domains are not independent of one another. On the contrary: individuals are nested within several levels of relational context, in which organizations occupy a significant role and interact with other domains. Building on this, the COR theory states that individuals feel well when they gather and retain resources from several domains of their lives. Organizations make no exception: positive personal and relational experiences at work likely contribute to employees’ general well-being [55]. This link is specifically interesting from an organization-based point of view. Studies in the field of positive psychology have indeed showed that when people acknowledge and savor their positive emotions, they are more likely to expand their cognitive and behavioral repertoires [103], with positive effects on performance and engagement at work [13,104]. 

Furthermore, the link between received compassion and life satisfaction is consistent with Seligman’s PERMA model, in which positive relationships account for a significant section of general well-being, together with positive emotions, engagement, meaning, and accomplishment [105]. In the model, these dimensions mutually influence to create a general sense of fulfillment and well-being. Consistently, previous studies on compassion suggest effects of sharing compassion among colleagues and supervisors that go above and beyond the mere having “positive relationships” [14,28]. Receiving compassion from others impacts other dimensions of the PERMA state, which could help understand our results. For example, it was linked to reduced suffering and a higher sense of comfort and positive emotions [29,33,42], as well as to a higher ability to put into perspective personal and others’ stressors and find proactive ways to regulate personal and others’ negative emotions (e.g., [106]). Further studies may expand current knowledge on the effects of compassion at work on several dimensions of personal and work-related well-being, even using the PERMA model of well-being, which was recently validated in the organizational field [107]. 

The COR theory and the related crossover model can provide meaningful suggestions about the mediating role of perceived school collective performance in the link between experienced compassion and life satisfaction. In the COR theory, the resources to gain and to retain encompass several aspects, from objects to money and from time to beliefs and mindsets [72], and can be shared across different life domains. The crossover model emerged from the systematization of the COR theory within organizations [72]. It states that individuals transmit psychological states from one another, whether directly or indirectly [108], so that the gathering of resources can be strengthened through the interactions [72]. In other words, colleagues contribute to building reciprocal realities at work, nurturing coworkers’ beliefs and mindsets about the job. It is likely that receiving compassion at work, as a deep form of connection and interaction, can foster the crossover effect to build up a sense of shared acknowledgment and management of tasks, contributing to building teachers’ ideas of performance. Building on the same model, because collective performance beliefs are resources in the COR theory, they are likely to contribute to participants’ satisfaction beliefs about life in general.

Finally, we did not confirm the expected mediating effect of burnout between experienced compassion and life satisfaction. Studies on emotional responses to events affirm that positive and negative emotions are independent. Indeed, they activate different biological and behavioral patterns and are independently regulated over time [109,110]. Although studies addressing their relationship report an inverse association, it is plausible to believe that, over time, the amounts of experienced positive and negative emotions do not relate to one another [109]. In other words, positive and negative emotions do not constitute the poles of a continuum. Thus, while studies on positive and negative emotions address the links between the two when explaining emotional reactions or beliefs related to emotions at school [111], this inverse association may disappear when addressing more complex constructs. Burnout, indeed, includes not only emotional but even physical and cognitive signs [112,113]. At the same time, compassion includes motivational, affective, and cognitive dimensions [114]. The multidimensional nature of the constructs may enrich the associations among the variables included in the study so that the burnout condition can coexist with the resources considered in the model while not being influenced by them.

## 5. Conclusions

Overall, the findings suggest a protective effect of received compassion at work on a work-related outcome (perceived collective performance) and a personal outcome (life satisfaction). Moreover, thanks to contributions from the broaden and build theory [115], the PERMA model [105], and the COR model [72], it is clear that organizations promoting supportive relationships impact employees’ well-being and performance. Compassionate behaviors, indeed, can be shown as a result of personal and organizational training [19,116,117] and other organizational actions. 

Concerning training, studies on healthcare professionals showed the promising effect of compassion-based training on several dimensions (e.g., stress, perceptions of organizational culture, personal and collective efficacy) and organizational actors (workers, leaders and managers, users) [19]. The main training objective is to help organizations build a compassionate culture so that employees feel recognized as individuals with personal needs, desires, and scopes, from one side. From the other, the whole workforce is managed with an understanding, participative, inclusive attitude, in which each member contributes to wider objectives [118,119]. Building on the literature that includes teachers in the helping professions [120], it is likely that this type of intervention benefits schools as well, with positive effects for teachers, principals, administrative staff, students, and their families. Previous studies on school management, indeed, suggest that school are typically characterized by distributed leadership, namely a form of leadership that emerges from individual conducts and relationship within the school staff [121,122,123]. According to these studies, leadership at schools emerges from interactions among people. This type of structure can constitute a fertile ground to insert compassion-based training, which aims to boost the value given to the experience and contribution of each employee.

A recent study showed that teachers who received an 8-week compassionate mind training program (CMT-T) improved their self-compassion ability and compassion for other skills. As a result, the authors pointed out that following the training, the teachers had developed a more kind, caring toward themselves and a nonjudgmental attitude toward their suffering, as well as a greater motivation to act compassionately to alleviate the other people’s suffering [124]. The conjunct effect on self- and other-oriented compassion is particularly interesting. According to studies on helping professionals’ compassion fatigue, indeed, workers with high compassion-related demands at work may suffer from burnout experiences linked with caring or suffer from secondary traumatic stress [125]. This is particularly true when they do not have adequate self-regulatory resources that could contribute to building stronger boundaries with their work experiences [126,127]. In this regard, the effect of the CMT-T is promising: while promoting compassion towards others and, consequently, the several positive effects mentioned in this paper, compassion-based training seem to allow for the building of self-protecting resources, thus preventing the risk of burning out. 

Regarding the effects of organizational actions on compassionate behaviors, it was shown that employees who perceive their organization as socially responsible are more prone to show compassionate behaviors toward colleagues, so employees’ positive perceptions of CSR actions boost compassionate behaviors at work [128]. Such findings suggest a cross-effect between the systemic level of organizational actions and employee-related beliefs and behaviors. Considering the cruciality of the organizational dimension to respond to the sociocultural changes imposed on schools in these times, it is likely that teachers’ satisfaction and commitment may benefit from organizational actions that foster a sense of belonging [48], and this, in turn, may foster their compassion and well-being. However, our work is not without limitations. Firstly, we used convenience sampling, and so our, participants do not represent the whole Italian teaching community, which may constitute an issue for the external validity of our data. Secondly, the study is cross-sectional, describing how the variables are linked in this sample, but it cannot be used for predictive purposes. A longitudinal study may better suit this type of objective and help clarify the role of burnout. Thirdly, this study involves only teachers, while schools as organizations include administrative staff members and other employees that support the teaching and learning processes (e.g., janitors and custodians). Future studies may introduce non-teaching school members to provide a more comprehensive view of these topics. Finally, based on our findings, future research may consider other subjective well-being outcomes besides life satisfaction. Furthermore, in addition to the compassion received at work, self-compassion could be tested to analyze whether a dispositional component can be significant in addition to an external component.

## Figures and Tables

**Table 1:** Correlations among study variables.

Variables	M	SD	1	2	3	4
1. Experienced compassion	3.644	0.093	-	−0.324 **	0.564 **	0.473 **
2. Burnout	3.200	0.441		-	−0.334 **	−0.440 **
3. Perceived school collective performance	3.547	0.740			-	0.547 **
4. Life satisfaction	4.901	1.310				-

Note. ** *p* < 0.01.

## Data Availability

Data are available upon request.
